# HATs与HDACs在肺癌上皮细胞间质转化中作用机制及其应用的研究进展

**DOI:** 10.3779/j.issn.1009-3419.2013.04.07

**Published:** 2013-04-20

**Affiliations:** 300052 天津，天津市肺癌转移与肿瘤微环境重点实验室，天津市肺癌研究所，天津医科大学总医院 Tianjin Key Laboratory of Lung Cancer Metastasis and Tumor Microenvironment, Tianjin Lung Cancer Institute, Tianjin Medical University General Hospital, Tianjin 300052, China

**Keywords:** 组蛋白乙酰转移酶, 组蛋白去乙酰化酶, 肺肿瘤, 上皮细胞间质化, Histone acetyltransferases, Histone deacetyltransferases, Lung neoplasms, Epithelial-mesenchymal transition

## Abstract

肺癌是危害我国人民健康与生命的重大疾病之一, 肺癌的复发和死亡多源于肿瘤转移。上皮细胞的间质化过程(epithelial-mesenchymal transition, EMT)是肺癌转移中的一个关键步骤, 此过程涉及E-cadherin表达下调, 并受到EMT转录因子调控。组蛋白乙酰转移酶(histone acetyltransferases, HATs)和组蛋白去乙酰化酶(histone deacetyltransferases, HDACs)是催化组蛋白乙酰化和去乙酰化的蛋白家族, 不仅在肿瘤进程中发挥重要功能, 近年来发现它们同样参与肺癌EMT过程。HATs与HDACs和某些EMT转录因子有相互作用。而且, 这些EMT转录因子的功能受乙酰化调控, 并影响肺癌EMT进程。本文将分别介绍HATs和HDACs参与肺癌EMT的作用机理, 从分子机制方面对它们之间的相互作用进行探讨, 并讨论HDAC抑制剂在抑制EMT和肺癌治疗方面的潜在应用价值, 以期为相关基础研究和临床实践提供借鉴。

## Abstract (en)

Lung cancer is one of the most common diseases that endanger health and life of people domestically.A number of recurrence and death of lung cancer originated from metastasis.As a key step in metastasis of lung cancer, epithelial to mesenchymal transition involved down-regulation of E-cadherin, as well as regulated by EMT transcription factors.HATs and HDACs is a protein family that catalyzes acetylation and deacetylation of histones.Not only they have vital functions in tumor pathogenesis, but also participate in the EMT of lung cancer.HATs and HDACs interact with certain EMT transcription factors.Moreover, the function of these EMT transcription factors may be regulated by acetylation, which has influence on EMT program in lung cancer.Therefore, this review introduces the event of HATs and HDACs function in EMT of lung cancer, and investigate the molecular mechanism of their interaction.Then, the potential of HDAC inhibitor utilization in the inhibition of EMT and lung cancer therapy were discussed, as to pave the way for the related basic research and clinical practice.

组蛋白乙酰转移酶(histone acetyltransferases, HATs)是一类参与基因表达调控的重要蛋白质家族, 其催化底物蛋白质的乙酰化修饰过程。组蛋白和非组蛋白都可作为HATs的底物, 其中组蛋白的乙酰化有利于打开致密的核小体结构, 活化基因转录的起始。与此相反, 组蛋白去乙酰化酶(histone deacetyltransferases, HDACs)催化相反过程, 从而使靶基因沉默。在细胞中, HATs和HDACs调控组蛋白的乙酰化水平, 使得靶基因的水平保持在动态平衡中。可乙酰化的非组蛋白包括转录因子、细胞骨架和信号通路蛋白等, 它们乙酰化后的功能也是多样的, 如转录激活、亚细胞定位、微管调控和细胞周期调控等。这些HATs的底物蛋白共同组成了体内的乙酰化蛋白网络, 对于细胞正常的生长发育、维持内环境稳态十分重要。某些重要信号途径中的HATs或HDACs产生突变, 将很可能导致疾病发生乃至癌变。

上皮细胞间质化(epithelial-mesenchymal transition, EMT)是上皮细胞在外界刺激或病理条件下, 细胞之间的连接弱化并失去极性, 进而具有间质细胞特征形态的过程。因此EMT在肿瘤上皮细胞转化为间质细胞的过程中发挥功能, 参与肿瘤的侵润与迁徙。研究发现, 细胞内某些转录因子参与调控EMT过程, 包括Snail、Slug、Twist、ZEB1/2等蛋白。Snail、Slug和ZEB通过直接抑制E-cadherin启动子活性来促进EMT过程, Twist等其它因子则间接抑制E-cadherin转录, 说明这些转录因子在EMT的调控中行使重要功能。

近年来, 有关HATs和HDACs家族蛋白参与肺癌EMT过程的报道逐渐增多。一类关注于p300、PCAF等HATs与EMT转录因子的相互作用及其对间质化过程的影响。某些EMT转录因子和HATs具有蛋白质相互作用, 甚至作为底物被乙酰化修饰, 初步阐明了HATs家族蛋白参与肿瘤EMT过程的调控机制。另一类则研究HDACs参与肺癌EMT的作用机理, 包括肺癌组织中HDACs表达的相关性研究, 以及HDACs与EMT转录因子相互作用抑制E-cadherin表达水平的机制等。还有报道通过HDACs的抑制剂(HDAC inhibitor, HDACi)处理细胞, 发现多种肺癌细胞中的HDACs被抑制后, E-cadherin表达上调, EMT转录因子被抑制, 进而使EMT过程受阻, 表明这一类药物具有影响EMT和临床治疗的潜在应用价值。据此, 本文分别从HATs、HDACs参与肺癌EMT的作用机制, 以及HDACi在肺癌治疗中的潜在应用等方面进行论述, 以期探讨HATs和HDACs调控肺癌EMT的分子机制和应用前景。

## 1 HATs参与肺癌EMT的作用机制

### 1.1 p300与肺癌EMT及其转录因子

近年来, 关于HATs家族蛋白尤其是p300和EMT关联性的研究逐渐增多^[1, 2]^。作为细胞内重要的HATs之一, p300参与多种肿瘤发生与进展过程, 其突变或失调导致多种疾病, 许多癌基因的表达也受到p300调控。

关于肺癌中p300参与EMT的分子作用机制, 目前已有初步了解。台湾一个研究小组^[3]^的结果表明, 肺癌迁徙中Runx2的水平上调有利于募集p300并促进Snail转录, 进一步实验发现其机制是Runx2通过募集p300增强组蛋白乙酰化, 继而使Snail表达上调, 说明p300可能在Snail诱导的EMT中发挥重要功能。还有研究^[4]^基于p300和Ⅰ型丝氨酸/苏氨酸激酶受体(serine/threonine kinase receptors Ⅰ, TβR Ⅰ)在细胞核中共定位的结果, 发现p300和TβR Ⅰ相互作用并激活Snail转录, 促进肿瘤细胞的侵袭。此外, 另一EMT转录因子Twist也证实与p300有相互作用, 而且Twist与p300的HAT结构域直接结合, 干扰其乙酰化组蛋白, 体外乙酰化实验亦证明这一结果。在肿瘤细胞中Twist可能通过抑制p300对组蛋白的乙酰化修饰, 促进上皮细胞的间质化过程^[5]^。Postigo等^[6]^发现ZEB1可能募集p300并促进转录激活, 使得ZEB1活性升高, 从而有利于EMT过程。生物信息学分析显示E-cadherin启动子含有潜在的p300结合位点, Liu等^[7]^通过染色质免疫共沉淀实验, 得到的研究结果确证了这一设想。即在p300与E-cadherin启动子结合的模型中, p300可能募集其它转录因子, 共同促进E-cadherin的表达水平。

### 1.2 PCAF与肺癌EMT及其转录因子

p300/CBP结合蛋白相关因子(p300/CBP-bingding protein-associated factor, PCAF)是另一种在人类和其它真核生物中保守存在的组蛋白乙酰转移酶, 同样具有乙酰化组蛋白的活性, 行使转录辅激活因子功能。Shiota等^[8]^对PCAF和Twist的相互作用进行研究, 发现PCAF可乙酰化Twist, 其底物Lys分别位于第73、76和77位。乙酰化促进Twist进入细胞核并促进其转录水平, 进而细胞的侵袭能力增强, 有利于EMT的发生。如将Twist的乙酰化Lys突变, 使其无法被PCAF乙酰化, 则阻碍Twist进入细胞核, 说明Twist的乙酰化修饰对其发挥促EMT功能是重要的。如敲除PCAF, 同样观察到肿瘤生长和侵袭受抑制。Hamamori的小组^[5]^证实了Twist同样抑制PCAF乙酰化组蛋白, 这种现象基于Twist的N端和PCAF的HAT结构域/溴区之间的相互作用, 因为N端缺失的Twist不能抑制PCAF的乙酰化功能。还有证据表明PCAF参与ZEB1调控miR-200水平的模型:一般情况下, ZEB1抑制miR-200c/141转录, 而PCAF和p300可与ZEB1在miR-200c/141启动子处组装成复合物, 同时PCAF乙酰化ZEB1, 使ZEB1由抑制物转变为激活因子, 促进miR-200c/141转录上调。这些结果说明ZEB1的活性是受乙酰化调节的, 暗示在肿瘤细胞中EMT转录因子功能受翻译后修饰调控可能具有一定普遍意义^[9]^。

### 1.3 其它HATs与EMT及其转录因子

CBP与p300在结构和功能上有较高的同源性, 在体内促进CREB介导的转录。在果蝇的胚胎发育中, 发现果蝇CBP可结合于Twist启动子并激活Twist表达, 与此相反, CBP突变体无法正常诱导Twist表达, 进而影响果蝇的胚胎发育^[10]^。Hlubek等^[11]^通过酵母双杂交实验证实, 组蛋白乙酰转移酶Tip60与ZEB蛋白有相互作用, 它们可能形成复合物, 共同起阻遏作用。如将Tip60敲除, 可使组蛋白乙酰化减弱, 并增强细胞对顺铂的敏感性^[12]^。虽然这些结果与肺癌细胞EMT并非直接关联, 但对于在肺癌中开展类似研究具有一定参考价值。

## 2 HDACs参与肺癌EMT的作用机制

随着对HATs在肺癌EMT中作用机制的深入研究, 关于HDACs与肺癌EMT关系的研究也为人们所关注。Han等^[13]^通过免疫组化和RT-PCR对非小细胞肺癌(non-small cell lung cancer, NSCLC)组织中的HDAC1和HDAC2的表达水平进行研究, 发现与正常肺组织相比, NSCLC组织中HDAC1和HDAC2的表达具有更高的相关性, 与分化程度和TNM分期也有一定关联。这一结果表明对于肺癌细胞的生长和分化, HDACs同样发挥重要作用。

有关HDACs在肺癌及肿瘤EMT中的作用机制, 以E-cadherin表达受HDACs抑制的分子机理研究较为清楚。Peinado等^[14]^发现Snail与HDAC1和HDAC2有相互作用, 而且Snail可募集它们结合E-cadherin启动子, 组成抑制复合物, 从而使组蛋白H3和H4去乙酰化, 抑制E-cadherin的转录起始, 最终有利于EMT过程。与此同时, 有研究小组同样观察到HDAC1的过表达导致E-cadherin启动子的活性减弱, 转染HDAC1的shRNA可有效抑制肿瘤细胞的转移, 进一步证实了HDAC1与侵袭性有关^[15]^。Tang等^[16]^的研究发现高热量饮食导致的E-cadherin降解和EMT过程中, HDAC表达水平也呈现上升趋势, 暗示其在间质化过程中具有一定普遍性。Snail自身的转录也受HDAC1抑制, 但p68的磷酸化使HDAC1从Snail启动子上解离, Snail水平随即上调, E-cadherin表达受阻而导致EMT发生^[17]^。此外, 一项在肺癌细胞HCC95中的研究^[18]^表明, 细胞中HDACs的水平上升可能源于miR-449a/b的调节, 从另一角度说明肺癌细胞中HDACs的调控可能是一个多层次、复杂的过程。

综上所述, HDACs在肺癌与肿瘤EMT中, 其表达水平与正常组织有差异。从分子机制来看, 它可与EMT转录因子相互作用并直接影响靶基因表达水平, 以达到沉默上皮细胞基因的目的, 导致间质化过程的发生。HDACs也可能通过其它途径影响肺癌细胞的生长分化。因此, HDACs具有作为抑制肺癌EMT与临床治疗靶点的潜在意义, 其应用前景值得深入探讨。

## 3 HDACi在肺癌治疗中具有潜在应用价值

如前文所述, HDACs在抑制肺癌E-cadherin表达水平和促进EMT过程中具有关键作用。据此, 将其作为治疗靶点, 用药物处理以期干扰HDACs行使功能, 不失为阻止肺癌细胞EMT和细胞迁徙、转移, 并应用于肺癌治疗的有效途径。目前, 已有HDAC抑制剂应用于肺癌细胞, 促进E-cadherin表达回复的报道, 为进一步将其研发为药物奠定了基础。Kakihana等^[19]^用多种HDACi处理NSCLC细胞系并检测细胞中EMT标志物的表达水平, 他们的结果表明MS-275上调E-cadherin的效果最为明显, 且诱导细胞凋亡和抑制NSCLC细胞生长。另一项对长期吸烟影响EMT的研究^[20]^中, 研究者发现MS-275抑制烟草浓缩物处理的A549细胞的迁徙与侵润, E-cadherin也有所上调。Mateen等^[21]^用TSA与silibinin处理H1299细胞, 观察到细胞中E-cadherin水平明显回复, 肿瘤细胞的侵润和迁徙能力也得到明显抑制。与此同时, ZEB1的表达亦受影响而下调, 说明TSA与silibinin具有协同抑制肺癌细胞EMT的作用。

鉴于HDACi本身具有促进组蛋白或底物蛋白乙酰化的功能, 所以此类药物在实际应用中, 也可能经由这一途径实现抑制肺癌细胞的效应。Singh等^[22]^用*Magnolia grandiflora*中提取的honokiol处理A549、H460等NSCLC细胞, 发现它具有抑制HDAC活性的功能并促进组蛋白乙酰化, 继而NSCLC的细胞活性明显降低。通过口服honokiol, 接种于裸鼠皮下的A549和H1299细胞生长也受到明显抑制。在NSCLC中silibinin可通过抑制HDAC上调组蛋白H3和H4的乙酰化水平, 与TSA共处理则导致G_2_/M阻滞和细胞凋亡, 并抑制异种移植肿瘤的生长, 表明HDACi与silibinin联用应该是一种安全有效的治疗NSCLC生长的用药方式^[23]^。

另一方面, 用HDACi与其它药物共处理肺癌细胞, 被认为可进一步发挥两种药物的协同抑制作用, 因而是HDACi应用于肺癌治疗的又一有效途径。在一篇最新报道中, 研究人员用一种新的HDACi-PTACH与17-DMAG(分子伴侣Hsp90的抑制剂)共同处理A549、H460和H1299细胞, 获得了比单药物处理更好的对肿瘤细胞迁徙的抑制效果^[24]^。Zhang等^[25]^观察到在培养的A549细胞中, 经TSA与化疗药物docetaxel或erlotinib共处理, 可上调α-tubulin的乙酰化水平、诱导细胞凋亡并抑制肿瘤细胞的增殖, 因此他们认为HDACi与化疗药物联用具有协同抑制肺癌细胞的功能。鉴于DNA甲基化在肿瘤转移中的重要功能, 将VPA和decitabine(一种DNA甲基转移酶抑制剂)共处理U1906和H2171细胞, 发现caspase-8在mRNA和蛋白水平均上调, 并使得这些小细胞肺癌细胞对肿瘤坏死因子相关的凋亡诱导配体(tumor necrosis factor-related apoptosis-inducing ligand, TRAIL)治疗变得敏感, 因而具有潜在临床价值^[26]^。

综合这些结果, 可知HDACi具有抑制肺癌细胞EMT的功能。它也可通过促进组蛋白乙酰化, 以及与其它药物共同使用来影响多种肺癌细胞的生长, 所以在肺癌临床治疗中具有较好的潜在应用前景。如对HDACi与其它药物联用的领域继续深入研究, 将使肺癌的治疗更加游刃有余。

## 4 问题与展望

肺癌作为危害我国人民健康与生命的重大疾病之一, 长期以来都是研究的热点领域。转移是导致肺癌难以治愈和死亡的重要因素, 它是一个复杂、多层次和多阶段的病理过程, 涉及抑癌基因突变、肿瘤相关蛋白表达水平改变、信号通路的活化等方面。其中, EMT过程是肺癌转移的重要步骤, 参与此过程的转录因子(如Snail和Twist)在调控此过程中起到关键作用。从功能上, 已知EMT转录因子抑制E-cadherin等蛋白的表达水平, 但其在肺癌中的详细作用机制尚不清楚。HATs和HDACs作为基因表达水平的重要调节因子, 控制着机体内环境的动态平衡。近年的研究发现它们同样参与上皮细胞的间质化进程, 可与某些EMT转录因子相互作用并使其乙酰化。而且, 这一研究方向在其它类型的癌症中同样有所报道^[27-31]^, 说明此领域有可能成为阐明EMT和肿瘤转移分子机制的新突破点。鉴于HDACs具有促进EMT的功能, 使用TSA等HDACi抑制其表达水平, 已被诸多研究^[7, 29, 32]^证实可有效抑制多种肿瘤细胞的EMT过程。与其它药物联用以强化对肿瘤细胞的抑制作用, 是HDACi应用于肺癌治疗的新兴领域。因此, 筛选具有较好抑制效果、副作用少的HDACs抑制剂, 利于为研发治疗肺癌或其它癌症转移的药物开拓新的方向。综上所述, 对HATs和HDACs在肺癌细胞EMT中作用机制的研究, 有助于丰富肺癌转移的分子生物学基础研究, 而HDACi对于EMT过程的有效抑制, 更预示着其在肺癌临床治疗中具有重要的潜在应用价值。
